# 

**Published:** 1923-11

**Authors:** 


### HOSPITAL and HEALTH

REVIEW

*Established 1886* *No.* 1868. *Vol. LXXII.*

*New Series.* Vol. II No. 26 *November,* 1923. Price Sixpence.

CONTENTS.

PAGE
An Overburdened Ministry . . . . . . 387
Nerves and Communications . . . . . . 388
Notes and Comments . . . . . . . . 389
Pure Food—Humane Slaughtering—Going Ratting—The Conquest of Liverpool—Pasteurisation of Milk—"The Lancet"—Popularising Health—Sobriety by Taxation—Life Assurance and Surgery—The Prevention of Gonorrhœal Ophthalmia—The World's Radium—Disadvantages of Aluminium Paint—Child Life in the Dominions—Patients of the Law—The Measurement of Intelligence—The Nature of Thirst.
A "Live Hospital" . . . . . . . 393
Prizegiving at the Middlesex Hospital . . . 394
Neglect of Minor Disease . . . . . . 394
National Health Insurance: A Momentous Decision . . . . . . . . . . 395
The Art of Living. By Miss H. S. Cooper Hodgson 397

PAGE
Hospital Tablets and Memorials . . . . . 398
The Co-ordination of Local Health Services: I.—Wanted, a Plea for the Public Health . 399
Popular Lectures on Cancer . . . . . 400
The Cost of Collection . . . . . . 401
The Highbury Hospital Fire . . . . . 401
Middlesex Hospital School: Portraits of Founders . . . . . . . . . 402
Financial Position of the London Hospitals . 404
Hospital Progress and Finance . . . . . 405
The High Grade Mental Defective . . . . 407
Correspondence . . . . . . . . 408
Homes for Aged Nurses . . . . . . 409
Reviews of Books . . . . . . . . 410
Points from Hospital Reports . . . . . 414
Recent Appointments . . . . . . . 414
Echoes of the Month . . . . . . . 415
Hospital and Health News . . . . . . 417

